# Role of Fcγ Receptor Mediated Inflammation in Immune Neuropathies

**DOI:** 10.4172/2155-9899.1000490

**Published:** 2017-03-08

**Authors:** Gang Zhang, Kazim A Sheikh

**Affiliations:** Department of Neurology, University of Texas Health Science Center at Houston, Houston, TX 77030, USA

## Introduction

Peripheral neuropathies are among the most common diseases encountered in clinical neurology. Autoimmunity and inflammation are implicated in a small but important group of peripheral nerve disorders because they are potentially treatable. These include the acute inflammatory neuropathies grouped under Guillain-Barré syndrome (GBS) and the chronic inflammatory demyelinating polyradiculoneuropathy (CIDP). GBS is the commonest cause of acute flaccid paralysis worldwide, and a life-threatening acute, monophasic, autoimmune peripheral neuropathy. GBS is subdivided into two major subtypes, i.e., axonal and demyelinating (acute inflammatory demyelinating polyradiculoneuropathy (AIDP)) variants [1-3]. CIDP and its variants are considered as the most common chronic inflammatory/immune neuropathy. It is believed that there is a temporal continuum between AIDP, the demyelinating form of GBS, and CIDP and some view CIDP as the chronic counterpart of AIDP form of GBS.

Understanding of the pathomechanisms of these inflammatory/immune neuropathies is incomplete but a large body of work favors synergism of cellular and humoral immune elements in the pathogenesis of these disorders [4-6]. A critical gap in our knowledge in this regard is that antigen specificity and nature of adaptive autoimmune responses are unknown, for the majority of neuropathic conditions grouped under GBS and CIDP, except axonal and Fisher variants of GBS. A key question in the context of inflammatory neuropathies is whether shared or final common pathway(s) of innate immune effectors, downstream of known or unknown adaptive nerve specific immune responses, which constitute endoneurial inflammation and mediate nerve (myelin and axonal) injury exist in individual inflammatory neuropathies grouped under GBS and CIDP and, if so, what are their key components?

Macrophages are an essential component of innate immunity, and a central regulator of inflammation. Two macrophage subpopulations, resident microglial cells and recruited hematogenous macrophages, respond and participate in the degenerative and regenerative processes after nerve injury. Growing body of research indicates that endoneurial inflammation orchestrated by macrophages is a critical component in autoimmune neuropathies [7-9]. Inflammatory cells in endoneurium, particularly macrophage recruitment is associated with early injury to nodes of Ranvier in GBS [10-12]. Macrophage-mediated myelin stripping and nodal and periaxonal macrophage-mediated attack on axons are pathognomonic features of demyelinating (AIDP) and axonal variants of GBS, respectively [9,13]. Monocyte-derived macrophages also appear to be important effectors of nerve injury in experimental studies, i.e., Experimental allergic neuritis (EAN) and AGA-mediated nerve injury models (see below). How macrophages mediate Schwann cell/myelin injury is not completely understood. The pathological studies favor both chemical (cell contact-independent) and phagocytic (cell contact-dependent) macrophage-mediated injury to myelin compartment in demyelinating variants of GBS but mechanisms and molecular actors involved in, perhaps, the dichotomous injury to Schwann cells/myelin are not completely understood.

There is strong evidence for the role of specific anti-glycan or ganglioside antibodies (AGAs) in the pathogenesis of axonal and Fisher forms of GBS [14-17]. Previous work has mostly concentrated on complement dependent cytotoxicity in AGAs-mediated nerve injury. However, adaptive humoral immunity (autoAbs or serum IgG) can use the powerful effector functions of cells of the innate immune system including monocytes/macrophages *via* Fc-gamma receptors (FcγRs) to induce target injury in infectious and autoimmune disorders [4-6]. In a variety IgG-dependent autoimmune models with tissue inflammation and injury evidence indicates a dominant role of immune complex (IC) and FcγRs *in vivo* [5,18] and a less prominent role of complement pathway [18,19]. The relevance of FcγRs in GBS is implied by some clinical studies showing that certain polymorphisms in activating FcγR genes are associated with risk of developing GBS and severity of the disease [20-22]. Meanwhile, the modulation of FcγRs had been considered as one mechanism of action of IVIg [4,23,24], a first line treatment for GBS and CIDP. IVIg can suppress the inflammatory responses *via* upregulation of the inhibitory FcγRIIB [4,23,25]. Notably, IVIG-responsive CIDP patients showed increases in FcγRIIB expression on B cells and monocytes [26,27]. Altogether, these observations raise the possibility that FcγRs mediated pro- and anti-inflammatory activities are critical in the development as well as treatment of the autoimmune neuropathic conditions, like GBS and CIDP, respectively.

FcγRs, classically described as activating FcγRs or inhibitory FcγRs, signal through immunoreceptor tyrosine activation or inhibitory motifs, respectively, are critical regulators of macrophage/microglia-mediated inflammation. In mice and humans, the family of FcγRs consists of three activating (FcγRI, III, IV in mice; FcγRIA, IIA, IIIA in humans) and one inhibitory (FcγRIIB in mice and humans) member [6,28]. Importantly, cells of innate immunity express activating and inhibitory FcγRs simultaneously, thereby setting a threshold for cell activation by IgG [5]. Among the three activating FcγRs, FcγRIII and FcγRIV in the mouse and FcγRIIA and FcγRIIIA in humans have a relatively lower affinity for soluble IgG and can only interact with Abs in the form of ICs [5,6] to prevent nonspecific activation of potent pro-inflammatory effector pathways by monomeric serum IgG. In contrast, FcγRI has a roughly 100-fold higher affinity for IgG subclasses (IgG2a in mice and IgG1/IgG3 in humans), enabling binding to monomeric IgG (Tables 1 and 2). Thus, high affinity FcγRI only plays a minor role in IgG-mediated injury to tissues [5,6]. Previous studies in various mouse models of immune diseases show that IgG2a and IgG2b isotypes mainly mediate injury *via* FcγRIV [29]. Occasionally, FcγRI and FcγRIII contribute to the activity of IgG2a suggesting that factors such as anatomical location, cytokine milieu, and effector-cell type contribute to specific FcγR usage in different inflammatory models [5]. In contrast mouse IgG1 mediates its inflammatory/cytotoxic effects exclusively *via* FcγRIII in various models of Ab-mediated tissue injury [30,31]. The critical step in triggering inflammation/effector cell response is initiated by crosslinking of FcγRs by IgG or ICs. This can occur either by interactions of low affinity, high avidity IgG ICs or of IgG opsonized cells with activation of FcγRs. Crosslinking of activating FcγRs induce a sustained calcium influx that participates in inflammation and cytotoxicity *via* degranulation, phagocytosis, ADCC, and release of cytokines and other proinflammatory mediators [5]. Cell activation initiated *via* activating FcγRs can be synergized by coengagement with other receptors including complement receptors. In contrast crosslinking of inhibitory FcγRIIB results in the arrest of these effector responses mediated by activating FcγRs, which is critical in maintaining balance between auto-immunity and tolerance.

## Evidence for the role of macrophages and FcγRs in experimental models of inflammatory neuropathies

EAN, a T-cell orchestrated model, has been used over last 50 years to study inflammatory demyelinating peripheral nerve injury in experimental animals. This model in its various versions recapitulates key clinical and pathological features of AIDP and CIDP. EAN is typically generated in laboratory animals by immunization with peripheral nerves, myelin, myelin proteins or their peptides [32]. The role of lymphocytic and monocytic inflammation in nerve fiber demyelination has been emphasized since the very first description of the model [33,34]. Pathological studies in this model show close relationship of monocyte/macrophages with demyelination including the presence of myelin debris in post-phagocytic monocytic cell populations, emphasizing these cells as immune effectors mediating myelin injury and clearance. Subsequent studies in EAN have confirmed the orchestrating role of lymphocytes in inducing nerve injury in adoptive EAN models. Ultrastructural studies on EAN nerves have highlighted the role of mononuclear cells and both mononuclear cellular contact-dependent and-independent mechanisms of demyelination have been implied. A number of studies have shown the effector role of macrophages in active EAN [35]. The essential role of macrophages in endoneurial inflammation and nerve fiber demyelination was established in adoptive EAN studies in which different macrophage-depletion strategies were employed [36]. Adoptive EAN is important as active EAN with myelin or myelin protein immunization include an induction phase of the autoimmune response to myelin antigens during which macrophages participate as antigen presenting cells in inducing lymphocytic adaptive immune responses. Adoptive EAN experimental paradigm precludes the antigen presenting role of macrophages as a mechanism of protection in this model. Macrophage-depletion strategies abrogating peripheral nerve demyelination in active and adoptive EAN models support the hypothesis, that macrophages are key components of endoneurial inflammation that mediate myelin/nerve fiber injury. Other studies in EAN support the idea that macrophages induce myelin injury by soluble effectors and myelin phagocytosis [37,38]. Specific monocyte/macrophage receptors that might mediate myelin injury in EAN have not been established. However, studies in experimental allergic encephalomyelitis, a related model of CNS demyelination, indicate that FcγRs also expressed by macrophage populations play a critical role in this model [39-41].

Our research focuses on elucidation of shared mechanisms (at cellular and molecular level) of nerve injury in antibody-mediated models of immune/inflammatory neuropathies to identify targets for novel therapies and testing novel therapies aimed at shared pathobiologic mechanisms underlying immune neuropathies in animal models. These preclinical studies are in the clinical context of axonal GBS, which is strongly associated with anti-ganglioside antibodies. In this regard we have focused on the role of monocyte-derived macrophage population and FcγRs as effectors of antibody-initiated endoneurial inflammation producing nerve injury in experimental models of immune neuropathies. We established two reproducible passive transfer animal models of AGAs-mediated nerve injury in the context of axonal GBS, including nerve crush model and spinal nerve transection model (modified Chung model), respectively. In nerve crush model, the nerve stumps distal to crush site have an inflammatory milieu due to recruitment of macrophages and virtually no BNB integrity. We can study the effects of different cellular and humoral immune elements on injured nerve fibers in nerve crush model. Whereas, modified Chung model allows us to focus on AGA’s effects on intact nerve fibers, because spinal nerve transection only causes degeneration of a small proportion of fibers that constitute sciatic nerve and its branches, majority of the fibers in sciatic nerve remain intact.

We showed that passive transfer of AGAs (experimental and human) impair nerve repair and severely inhibit axon regeneration, and ICs formed by AGA and its target antigen is required for the inhibition in nerve crush model [19,42,43]. These experimental findings echo the clinical association of AGAs and poor recovery in GBS. Moreover, the finding that AGAs mediated-inhibition of axonal regeneration was abolished in animals lacking all activating FcγRs confirm the pivotal role of activating FcγRs. Further studies in mutant mice deficient in individual activating receptors show that FcγRIII was the dominant activating FcγR involved in AGA (a mouse IgG2b mAb) mediated-inhibition. We previously demonstrated that hematogenous macrophages were rapidly recruited from the circulation and infiltrated the lesion site after peripheral nerve injury [44]. The role of macrophage/microglial cells in Ab-mediated inhibition of axon regeneration was assessed in crush model using macrophage colony-stimulating factor (M-CSF)-deficient osteopetrotic (B6C3Fe *a/a-Csf1*^op/op^) mice (*op/op*). In these mice, monocytes, tissue macrophages and osteoclasts are deficient. Our studies in *op/op* mice confirmed the involvement of macrophages in AGA-mediated pathological effects in this model [43]. In order to dissect the contribution of FcγRs-bearing circulating macrophage and FcγRs-expressing resident endoneurial glial cells, a nerve grafting study, in which wild type (WT) or activating FcγRs-null (*Fcer1g*−/−) nerve segments (donors) were transplanted in background matched WT or *Fcer1g*−/− (hosts) mice, was carried out [43]. We found that there is significantly more axon regeneration in WT or FcγRs-null grafts implanted in FcγRs-null hosts, compared to WT or FcγRs-null grafts implanted in WT hosts. These studies show that the macrophages recruited from the circulation of the host animals are the dominant cell type and endogenous nerve glia have minor contribution to the activating FcγRs-mediated inflammation and Ab-mediated inhibition of axon regeneration. A fundamental principle learned from these studies was that inflammatory milieu, primarily consisting of activated FcγR-bearing macrophage/microglia are critical mediators of Ab-mediated nerve injury. These data demonstrate that the passive transfer of AGAs can induce neuropathic injury but endoneurial inflammation particularly macrophages bearing Fcγ receptors are necessary for antibody-mediated pathogenicity.

**Figure 1:**
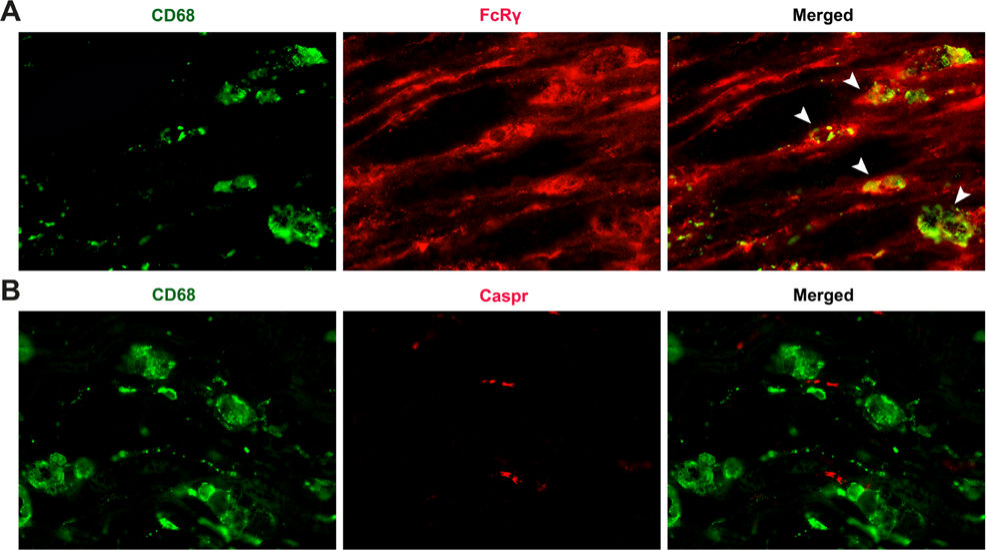
A) GBS nerve double labeled for CD68 (green) and Fcγ common chain (red). There is upregulation of FcγRs in macrophages (arrowheads). B) GBS nerve double labeled for CD68 (green) and Caspr (red) showing macrophages adjacent to widened nodes of Ranvier.

Our studies using modified Chung model indicate that AGAs induce sequential nodal (early) and then axonal (late) injury of intact myelinated nerve fibers and activated macrophages expressing activated FcγRs were found adjacent to widened nodes recapitulating pathologic features of human disease [45]. Importantly, this experimental neuropathy correlates with injury-related upregulation of activating FcγRs in resident endoneurial and recruited (macrophages) glial cells in the nerves. There is significant amelioration in AGA mediated-nodal and axonal injury in macrophage deficient (*op/op*) mice in this model. Notably, activating FcγRs-null (*Fcer1g*−/−) animals are completely protected from Ab-mediated nodal and axonal injury in this model. These studies provide further experimental evidence of the role of macrophages and activating FcγRs mediated-inflammation in Ab-mediated experimental models of autoimmune neuropathies. In line with these animal studies, we also found that macrophages and Schwann cells do express FcγR common chain in the nerves of patients with axonal GBS [43]. The FcγR common chain expression was significantly upregulated in GBS nerves compared to controls and activated macrophages expressing activating FcγRs were found adjacent to widened nodes in axonal GBS patient nerves (Figure 1).

## Concluding Remarks

Based on above clinical and experimental observations, we hypothesize that macrophages and activating FcγRs are innate immune effectors that partly constitute the final common executionary pathway of nerve injury in inflammatory neuropathies. A number of critical issues regarding this putative final common pathway of inflammatory nerve injury in the endoneurium remain to be elucidated. How endoneurial inflammation and macrophage recruitment is accomplished in AIDP and CIDP cases without prominent T cell inflammation and in axonal forms of GBS? Could peripheral nerve glial cells (including microglia and Schwann cells) be activated by signaling molecules entering the endoneurium from systemic immune compartment and subsequently creating inflammatory milieu in the endoneurium (including macrophage recruitment)? Our studies in the modified Chung model support that partial nerve injury generates endoneurial signals that compromise the integrity of blood-nerve barrier, recruit macrophages from circulation, and set up inflammatory milieu locally in the nerve. Moreover, our experimental studies in the context of anti-ganglioside antibodies and axonal injury indicate that activating FcγRs on macrophage populations are key molecular effectors mediating nerve injury. Whether the macrophage and activating FcγRs interactions with immune complexes formed on nerve fibers are random or other innate immune effectors (such as complement activation products) aid as chemoattractants for macrophage trafficking within endoneurium to specific sites along the nerve fibers is not known. What are the kinetics and evolving phenotype(s) (resting, pro-inflammatory, anti-inflammatory, or in between these polarized states) of macrophage/microglial cells in the endoneurial compartment of intact, injured, and diseased nerves? What are the molecular effectors and downstream pathways after IC formation and its interactions with macrophage/microglial populations? It is believed that multiple inflammatory pathways (with cellular and noncelluar inflammatory elements) in the endoneurium can produce similar nodal and axonal injury in experimental models as suggested by our work and a series of studies focusing on AGAs and complement [43,45-47]. In summary, understanding the endoneurial cellular and molecular inflammatory mechanisms that mediate nerve fiber injury could help identify novel targets for drug development in inflammatory and autoimmune neuropathies.

## Figures and Tables

**Table 1:** Affinity of different mice IgG isotypes to mouse FcγRs.

	FcγRI	FcγRIIB	FcγRIII	FcγRIV
IgG1	NB	3.3 × 10^6^	3.1 × 10^5^	NB
IgG2a	1.8 × 10^8^	0.4 × 10^6^	6.8 × 10^5^	2.9 × 10^7^
IgG2b	NB	2.2 × 10^6^	6.4 × 10^5^	1.7 × 10^7^
IgG3	NB	NB	NB	NB

NB: No binding

**Table 2:** Affinity of different human IgG isotypes to human FcγRs.

	FcγRI	FcγRIIA	FcγRIIB	FcγRIIIA	FcγRIIIB
IgG1	9.1 × 10^8^	5.2 × 10^5^	2 × 10^5^	2.2 × 10^7^	<10^7^
IgG2	NB	4.5 × 10^5^	0.2 × 10^5^	0.7 × 10^5^	NB
IgG3	6.1 × 10^7^	8.9 × 10^5^	1.7 × 10^5^	2.0 × 10^7^	<10^7^
IgG4	3.4 × 10^7^	1.7 × 10^5^	2 × 10^5^	2.5 × 10^5^	NB

NB: No binding
